# Ethyl 2-[1-(3-methyl­but­yl)-4-phenyl-1*H*-1,2,3-triazol-5-yl]-2-oxo­acetate

**DOI:** 10.1107/S1600536813030420

**Published:** 2013-11-13

**Authors:** Muhammad Naeem Ahmed, Khawaja Ansar Yasin, M. Nawaz Tahir, Muhammad Hafeez, Shahid Aziz

**Affiliations:** a Department of Chemistry, The University of Azad Jammu and Kashmir, Muzaffarabad 13100, Pakistan; b University of Sargodha, Department of Physics, Sargodha, Pakistan

## Abstract

In the title compound, C_17_H_21_N_3_O_3_, the non-planar (r.m.s. deviation = 0.212 Å) ethyl (oxo)acetate group is oriented towards the phenyl substituent. The triazole and benzene rings are twisted with respect to each other, making a dihedral angle of 41.69 (6)°. In the crystal, mol­ecules are arranged into centrosymmetric *R*_2_^2^(10) dimers *via* pairs of C—H⋯O inter­actions involving the ethyl (oxo)acetate groups. In addition, the triazole rings show π–π stacking inter­actions, with their centroids at a distance of 3.745 (2) Å.

## Related literature

For the biological activity of 1,4,5-trisubstituted 1,2,3-triazoles, see: Siddiqi & Ahsan (2010 ▶); Siddiqi *et al.* (2011 ▶). For the synthesis, see: Wang *et al.* (2013 ▶). For graph-set notation, see: Bernstein *et al.* (1995 ▶).
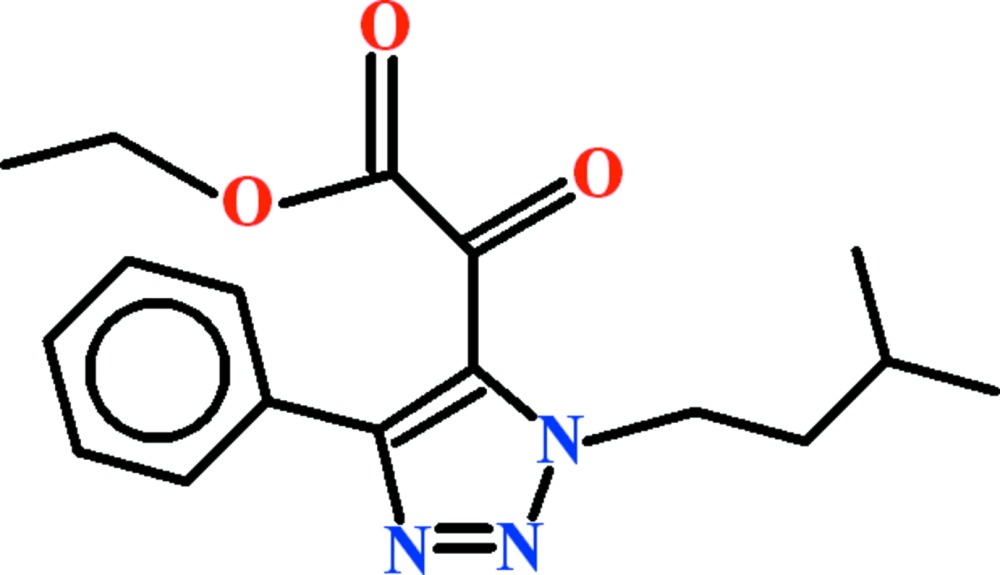


## Experimental

### 

#### Crystal data

C_17_H_21_N_3_O_3_
*M* *_r_* = 315.37
Triclinic, 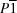

*a* = 8.1710 (8) Å
*b* = 10.0684 (9) Å
*c* = 10.6066 (10) Å
α = 98.331 (3)°
β = 94.220 (3)°
γ = 95.367 (3)°
*V* = 856.23 (14) Å^3^
*Z* = 2
Mo *K*α radiation
μ = 0.09 mm^−1^
*T* = 296 K
0.32 × 0.25 × 0.21 mm

#### Data collection

Bruker Kappa APEXII CCD diffractometer
Absorption correction: multi-scan (*SADABS*; Bruker, 2005 ▶) *T*_min_ = 0.973, *T*_max_ = 0.982
11605 measured reflections
4189 independent reflections
3196 reflections with *I* > 2σ(*I*)
*R*_int_ = 0.021

#### Refinement

*R*[*F* ^2^ > 2σ(*F* ^2^)] = 0.047
*wR*(*F* ^2^) = 0.156
*S* = 1.06
4189 reflections
211 parameters
H-atom parameters constrained
Δρ_max_ = 0.23 e Å^−3^
Δρ_min_ = −0.18 e Å^−3^

### 

Data collection: *APEX2* (Bruker, 2007 ▶); cell refinement: *SAINT* (Bruker, 2007 ▶); data reduction: *SAINT*; program(s) used to solve structure: *SHELXS97* (Sheldrick, 2008 ▶); program(s) used to refine structure: *SHELXL97* (Sheldrick, 2008 ▶); molecular graphics: *ORTEP-3 for Windows* (Farrugia, 2012 ▶) and *PLATON* (Spek, 2009 ▶); software used to prepare material for publication: *WinGX* (Farrugia, 2012 ▶) and *PLATON* (Spek, 2009 ▶).

## Supplementary Material

Crystal structure: contains datablock(s) global, I. DOI: 10.1107/S1600536813030420/gk2593sup1.cif

Structure factors: contains datablock(s) I. DOI: 10.1107/S1600536813030420/gk2593Isup2.hkl

Supplementary material file. DOI: 10.1107/S1600536813030420/gk2593Isup3.cml

Additional supplementary materials:  crystallographic information; 3D view; checkCIF report

## Figures and Tables

**Table 1:** Hydrogen-bond geometry (Å, °)

*D*—H⋯*A*	*D*—H	H⋯*A*	*D*⋯*A*	*D*—H⋯*A*
C11—H11*B*⋯O2^i^	0.97	2.59	3.338 (2)	134

Symmetry code: (i) 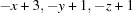.

**Fig. 1.**
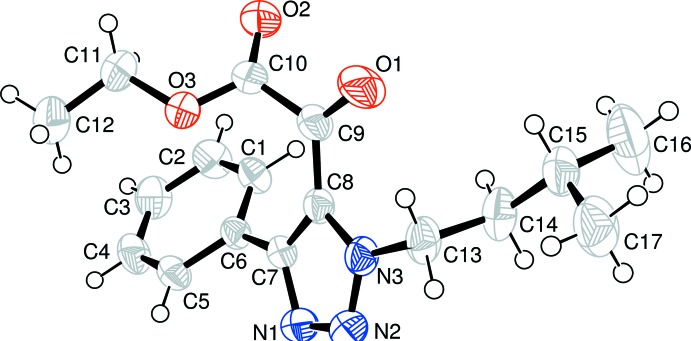
Molecular structure of the title compound. The displacement ellipsoids are drawn at the 50% probability level. H-atoms are shown by small circles of arbitrary radii.

**Fig. 2.**
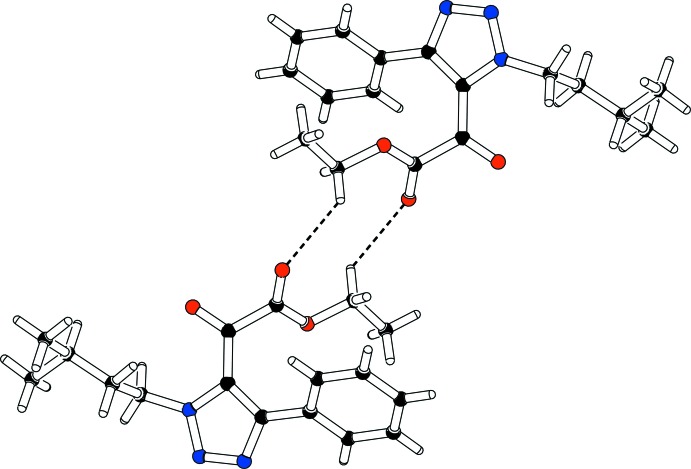
The dimers formed via C-H···O inteactions (PLATON: Spek, 2009).

## supplementary crystallographic information

### 1. Comment

1,4,5-Trisubstituted 1,2,3-triazoles are important compounds due to their diverse biological activities including antibacterial, antifungal, antimalarial, antiviral, anticonvulsant, antidepressant and anticancer (Siddiqi & Ahsan, 2010; Siddiqi *et al.*, 2011). Taking the biological activity of 1,2,3-triazole derivatives into account some novel 1, 2, 3-triazoles have been designed and synthesized. Here we report the crystal structure of title compound (Fig. 1).

The benzene ring A (C1–C6) and the triazol ring B (C7/C8/N1/N2/N3) are planar with r. m. s. deviations of 0.0062 Å and 0.0066 Å, respectively. The dihedral angle between A/B is 41.69 (6)°. The intermolecular C—H···O hydrogen bond (Table 1, Fig. 2) generates centrosymmetric *R*_2_^2^(10) motif (Bernstein *et al.*, 1995) and molecules form dimers. There is also π–π interaction between the triazole rings with their centroids at a distance of 3.745 (2) Å [*Cg*—*Cg*^i^: i = 2 - *x*, 1 - *y*, -*z*, where *Cg* is the centroid of triazol ring].

### 2. Experimental

To a suspension of 1-azido-3-methylbutane (0.068 g, 0.6 mmol) and 1-copper(I)phenylethyne (0.082 g, 0.5 mmol) in chlorobenzene (1 ml) was added ethyl chloro(oxo)acetate (0.068 g, 0.5 mmol). The resultant mixture was stirred at room temperature for 4 h and then passed through a column [silica gel, 10% EtOAc in petroleum ether (333–363 K)] to give the title compound as a white solid (yield 89%, m.p. 340–342 K).Crystals suitable for crystallographic study were grown by slow evaporation of an ethanol solution at room temperature (Wang *et al.*, 2013).

### 3. Refinement

The H atoms were positioned geometrically (C–H = 0.93–0.98 Å) and refined as riding on their carriers with *U*_iso_(H) = *xU*_eq_(C), where *x* = 1.5 for methyl and *x* = 1.2 for other H-atoms.

### Crystal data

**Table:**

C_17_H_21_N_3_O_3_	*Z* = 2
*M**_r_* = 315.37	*F*(000) = 336
Triclinic, *P*1	*D*_x_ = 1.223 Mg m^−^^3^
*a* = 8.1710 (8) Å	Mo *K*α radiation, λ = 0.71073 Å
*b* = 10.0684 (9) Å	Cell parameters from 3196 reflections
*c* = 10.6066 (10) Å	θ = 2.0–28.4°
α = 98.331 (3)°	µ = 0.09 mm^−^^1^
β = 94.220 (3)°	*T* = 296 K
γ = 95.367 (3)°	Block, colorless
*V* = 856.23 (14) Å^3^	0.32 × 0.25 × 0.21 mm

### Data collection

**Table:**

Bruker Kappa APEXII CCD diffractometer	4189 independent reflections
Radiation source: fine-focus sealed tube	3196 reflections with *I* > 2σ(*I*)
Graphite monochromator	*R*_int_ = 0.021
Detector resolution: 7.50 pixels mm^-1^	θ_max_ = 28.4°, θ_min_ = 2.0°
ω scans	*h* = −10→10
Absorption correction: multi-scan (*SADABS*; Bruker, 2005)	*k* = −13→13
*T*_min_ = 0.973, *T*_max_ = 0.982	*l* = −8→14
11605 measured reflections	

### Refinement

**Table:**

Refinement on *F*^2^	Primary atom site location: structure-invariant direct methods
Least-squares matrix: full	Secondary atom site location: difference Fourier map
*R*[*F*^2^ > 2σ(*F*^2^)] = 0.047	Hydrogen site location: inferred from neighbouring sites
*wR*(*F*^2^) = 0.156	H-atom parameters constrained
*S* = 1.06	*w* = 1/[σ^2^(*F*_o_^2^) + (0.0847*P*)^2^ + 0.1314*P*] where *P* = (*F*_o_^2^ + 2*F*_c_^2^)/3
4189 reflections	(Δ/σ)_max_ < 0.001
211 parameters	Δρ_max_ = 0.23 e Å^−^^3^
0 restraints	Δρ_min_ = −0.18 e Å^−^^3^

### Special details

**Table:**

Geometry. All esds (except the esd in the dihedral angle between two l.s. planes) are estimated using the full covariance matrix. The cell esds are taken into account individually in the estimation of esds in distances, angles and torsion angles; correlations between esds in cell parameters are only used when they are defined by crystal symmetry. An approximate (isotropic) treatment of cell esds is used for estimating esds involving l.s. planes.
Refinement. Refinement of *F*^2^ against ALL reflections. The weighted *R*-factor *wR* and goodness of fit *S* are based on *F*^2^, conventional *R*-factors *R* are based on *F*, with *F* set to zero for negative *F*^2^. The threshold expression of *F*^2^ > σ(*F*^2^) is used only for calculating *R*-factors(gt) *etc*. and is not relevant to the choice of reflections for refinement. *R*-factors based on *F*^2^ are statistically about twice as large as those based on *F*, and *R*- factors based on ALL data will be even larger.

### Fractional atomic coordinates and isotropic or equivalent isotropic displacement parameters (Å^2^)

**Table:**

	*x*	*y*	*z*	*U*_iso_*/*U*_eq_	
O1	1.02397 (18)	0.67468 (12)	0.33208 (13)	0.0700 (4)	
O2	1.21537 (16)	0.52473 (15)	0.48236 (11)	0.0699 (4)	
O3	1.28070 (12)	0.43994 (10)	0.28644 (10)	0.0466 (3)	
N1	0.82997 (15)	0.30393 (13)	0.04196 (12)	0.0477 (3)	
N2	0.78007 (16)	0.41791 (14)	0.01373 (12)	0.0510 (3)	
N3	0.84537 (14)	0.51779 (12)	0.10506 (11)	0.0423 (3)	
C1	0.99415 (18)	0.22438 (14)	0.34572 (14)	0.0440 (3)	
H1	0.9492	0.2933	0.3956	0.053*	
C2	1.0607 (2)	0.12404 (16)	0.40227 (16)	0.0533 (4)	
H2	1.0625	0.1265	0.4904	0.064*	
C3	1.1249 (2)	0.01966 (16)	0.32834 (17)	0.0554 (4)	
H3	1.1698	−0.0476	0.3669	0.067*	
C4	1.1224 (2)	0.01532 (15)	0.19828 (16)	0.0513 (4)	
H4	1.1642	−0.0556	0.1488	0.062*	
C5	1.05780 (18)	0.11619 (14)	0.14049 (14)	0.0436 (3)	
H5	1.0568	0.1130	0.0524	0.052*	
C6	0.99416 (16)	0.22261 (12)	0.21412 (13)	0.0363 (3)	
C7	0.92856 (16)	0.33138 (13)	0.15338 (12)	0.0366 (3)	
C8	0.94279 (16)	0.46949 (13)	0.19486 (12)	0.0371 (3)	
C9	1.04460 (18)	0.55757 (14)	0.29903 (14)	0.0434 (3)	
C10	1.18948 (18)	0.50382 (15)	0.36822 (14)	0.0450 (3)	
C11	1.4239 (2)	0.3868 (2)	0.34301 (17)	0.0611 (4)	
H11A	1.3911	0.3339	0.4082	0.073*	
H11B	1.5047	0.4605	0.3827	0.073*	
C12	1.4958 (2)	0.3015 (2)	0.2411 (2)	0.0755 (6)	
H12A	1.4173	0.2259	0.2058	0.113*	
H12B	1.5939	0.2696	0.2760	0.113*	
H12C	1.5230	0.3534	0.1750	0.113*	
C13	0.7973 (2)	0.65445 (16)	0.10329 (16)	0.0499 (4)	
H13A	0.7548	0.6630	0.0174	0.060*	
H13B	0.8935	0.7198	0.1274	0.060*	
C14	0.6661 (2)	0.68401 (19)	0.19540 (19)	0.0588 (4)	
H14A	0.6940	0.6483	0.2735	0.071*	
H14B	0.5608	0.6375	0.1573	0.071*	
C15	0.6481 (2)	0.8346 (2)	0.22911 (18)	0.0639 (5)	
H15	0.7564	0.8809	0.2636	0.077*	
C16	0.5290 (4)	0.8555 (3)	0.3333 (3)	0.1128 (11)	
H16A	0.5197	0.9501	0.3558	0.169*	
H16B	0.5701	0.8201	0.4074	0.169*	
H16C	0.4224	0.8093	0.3020	0.169*	
C17	0.5911 (3)	0.8960 (2)	0.1140 (2)	0.0793 (6)	
H17A	0.4850	0.8518	0.0786	0.119*	
H17B	0.6691	0.8847	0.0509	0.119*	
H17C	0.5829	0.9904	0.1394	0.119*	

### Atomic displacement parameters (Å^2^)

**Table:**

	*U*^11^	*U*^22^	*U*^33^	*U*^12^	*U*^13^	*U*^23^
O1	0.0919 (9)	0.0403 (6)	0.0726 (8)	0.0232 (6)	−0.0114 (7)	−0.0099 (5)
O2	0.0643 (7)	0.0953 (10)	0.0446 (6)	0.0281 (7)	−0.0090 (5)	−0.0131 (6)
O3	0.0415 (5)	0.0511 (6)	0.0460 (5)	0.0120 (4)	−0.0003 (4)	0.0009 (4)
N1	0.0489 (7)	0.0491 (7)	0.0431 (7)	0.0109 (5)	−0.0040 (5)	0.0006 (5)
N2	0.0509 (7)	0.0575 (8)	0.0442 (7)	0.0152 (6)	−0.0049 (5)	0.0045 (6)
N3	0.0432 (6)	0.0440 (6)	0.0428 (6)	0.0167 (5)	0.0026 (5)	0.0095 (5)
C1	0.0544 (8)	0.0363 (7)	0.0429 (7)	0.0117 (6)	0.0101 (6)	0.0037 (5)
C2	0.0700 (10)	0.0466 (8)	0.0471 (8)	0.0130 (7)	0.0080 (7)	0.0136 (6)
C3	0.0622 (9)	0.0414 (8)	0.0678 (10)	0.0170 (7)	0.0071 (8)	0.0173 (7)
C4	0.0579 (9)	0.0342 (7)	0.0631 (10)	0.0159 (6)	0.0125 (7)	0.0010 (6)
C5	0.0511 (8)	0.0343 (7)	0.0439 (7)	0.0078 (6)	0.0074 (6)	−0.0027 (5)
C6	0.0379 (6)	0.0292 (6)	0.0412 (7)	0.0068 (5)	0.0043 (5)	0.0004 (5)
C7	0.0368 (6)	0.0365 (6)	0.0363 (6)	0.0101 (5)	0.0038 (5)	0.0005 (5)
C8	0.0380 (6)	0.0366 (7)	0.0383 (6)	0.0128 (5)	0.0035 (5)	0.0053 (5)
C9	0.0485 (7)	0.0357 (7)	0.0451 (7)	0.0111 (6)	0.0019 (6)	0.0003 (5)
C10	0.0434 (7)	0.0426 (7)	0.0450 (8)	0.0072 (6)	−0.0027 (6)	−0.0046 (6)
C11	0.0444 (8)	0.0788 (12)	0.0589 (10)	0.0218 (8)	−0.0053 (7)	0.0015 (8)
C12	0.0546 (10)	0.0810 (13)	0.0877 (14)	0.0252 (9)	0.0058 (10)	−0.0090 (11)
C13	0.0537 (8)	0.0490 (8)	0.0548 (9)	0.0249 (7)	0.0094 (7)	0.0193 (7)
C14	0.0534 (9)	0.0615 (10)	0.0717 (11)	0.0276 (8)	0.0182 (8)	0.0240 (8)
C15	0.0625 (10)	0.0658 (11)	0.0666 (11)	0.0318 (9)	0.0047 (8)	0.0055 (8)
C16	0.137 (2)	0.129 (2)	0.0923 (18)	0.086 (2)	0.0459 (17)	0.0216 (16)
C17	0.0965 (15)	0.0644 (12)	0.0859 (14)	0.0416 (11)	0.0122 (12)	0.0181 (10)

### Geometric parameters (Å, º)

**Table:**

O1—C9	1.2115 (17)	C9—C10	1.530 (2)
O2—C10	1.1978 (19)	C11—C12	1.473 (2)
O3—C10	1.3209 (17)	C11—H11A	0.9700
O3—C11	1.4565 (18)	C11—H11B	0.9700
N1—N2	1.3199 (18)	C12—H12A	0.9600
N1—C7	1.3585 (18)	C12—H12B	0.9600
N2—N3	1.3325 (18)	C12—H12C	0.9600
N3—C8	1.3684 (17)	C13—C14	1.524 (2)
N3—C13	1.4680 (18)	C13—H13A	0.9700
C1—C2	1.381 (2)	C13—H13B	0.9700
C1—C6	1.3934 (19)	C14—C15	1.530 (2)
C1—H1	0.9300	C14—H14A	0.9700
C2—C3	1.385 (2)	C14—H14B	0.9700
C2—H2	0.9300	C15—C17	1.509 (3)
C3—C4	1.373 (2)	C15—C16	1.530 (3)
C3—H3	0.9300	C15—H15	0.9800
C4—C5	1.385 (2)	C16—H16A	0.9600
C4—H4	0.9300	C16—H16B	0.9600
C5—C6	1.3964 (17)	C16—H16C	0.9600
C5—H5	0.9300	C17—H17A	0.9600
C6—C7	1.4714 (17)	C17—H17B	0.9600
C7—C8	1.3877 (18)	C17—H17C	0.9600
C8—C9	1.464 (2)		
			
C10—O3—C11	115.68 (12)	O3—C11—H11B	110.0
N2—N1—C7	108.62 (12)	C12—C11—H11B	110.0
N1—N2—N3	108.25 (11)	H11A—C11—H11B	108.4
N2—N3—C8	110.66 (11)	C11—C12—H12A	109.5
N2—N3—C13	119.48 (12)	C11—C12—H12B	109.5
C8—N3—C13	129.67 (13)	H12A—C12—H12B	109.5
C2—C1—C6	120.11 (13)	C11—C12—H12C	109.5
C2—C1—H1	119.9	H12A—C12—H12C	109.5
C6—C1—H1	119.9	H12B—C12—H12C	109.5
C1—C2—C3	120.28 (15)	N3—C13—C14	110.78 (13)
C1—C2—H2	119.9	N3—C13—H13A	109.5
C3—C2—H2	119.9	C14—C13—H13A	109.5
C4—C3—C2	120.11 (14)	N3—C13—H13B	109.5
C4—C3—H3	119.9	C14—C13—H13B	109.5
C2—C3—H3	119.9	H13A—C13—H13B	108.1
C3—C4—C5	120.21 (13)	C13—C14—C15	113.33 (15)
C3—C4—H4	119.9	C13—C14—H14A	108.9
C5—C4—H4	119.9	C15—C14—H14A	108.9
C4—C5—C6	120.22 (14)	C13—C14—H14B	108.9
C4—C5—H5	119.9	C15—C14—H14B	108.9
C6—C5—H5	119.9	H14A—C14—H14B	107.7
C1—C6—C5	119.05 (12)	C17—C15—C14	112.37 (17)
C1—C6—C7	120.60 (11)	C17—C15—C16	110.58 (17)
C5—C6—C7	120.36 (12)	C14—C15—C16	109.5 (2)
N1—C7—C8	108.50 (11)	C17—C15—H15	108.1
N1—C7—C6	121.22 (12)	C14—C15—H15	108.1
C8—C7—C6	130.19 (12)	C16—C15—H15	108.1
N3—C8—C7	103.94 (12)	C15—C16—H16A	109.5
N3—C8—C9	122.89 (12)	C15—C16—H16B	109.5
C7—C8—C9	132.91 (12)	H16A—C16—H16B	109.5
O1—C9—C8	123.30 (13)	C15—C16—H16C	109.5
O1—C9—C10	116.91 (13)	H16A—C16—H16C	109.5
C8—C9—C10	119.75 (12)	H16B—C16—H16C	109.5
O2—C10—O3	126.60 (14)	C15—C17—H17A	109.5
O2—C10—C9	121.89 (14)	C15—C17—H17B	109.5
O3—C10—C9	111.44 (12)	H17A—C17—H17B	109.5
O3—C11—C12	108.46 (14)	C15—C17—H17C	109.5
O3—C11—H11A	110.0	H17A—C17—H17C	109.5
C12—C11—H11A	110.0	H17B—C17—H17C	109.5
			
C7—N1—N2—N3	0.13 (16)	N1—C7—C8—N3	1.65 (15)
N1—N2—N3—C8	0.96 (16)	C6—C7—C8—N3	−174.92 (13)
N1—N2—N3—C13	−174.42 (12)	N1—C7—C8—C9	−172.46 (15)
C6—C1—C2—C3	−1.3 (2)	C6—C7—C8—C9	11.0 (2)
C1—C2—C3—C4	−0.1 (3)	N3—C8—C9—O1	17.4 (2)
C2—C3—C4—C5	0.9 (3)	C7—C8—C9—O1	−169.39 (16)
C3—C4—C5—C6	−0.3 (2)	N3—C8—C9—C10	−160.12 (13)
C2—C1—C6—C5	1.9 (2)	C7—C8—C9—C10	13.1 (2)
C2—C1—C6—C7	−177.97 (14)	C11—O3—C10—O2	2.0 (2)
C4—C5—C6—C1	−1.1 (2)	C11—O3—C10—C9	178.99 (14)
C4—C5—C6—C7	178.75 (13)	O1—C9—C10—O2	46.5 (2)
N2—N1—C7—C8	−1.15 (16)	C8—C9—C10—O2	−135.78 (17)
N2—N1—C7—C6	175.78 (12)	O1—C9—C10—O3	−130.63 (16)
C1—C6—C7—N1	−136.58 (14)	C8—C9—C10—O3	47.07 (19)
C5—C6—C7—N1	43.59 (19)	C10—O3—C11—C12	171.29 (15)
C1—C6—C7—C8	39.6 (2)	N2—N3—C13—C14	99.01 (17)
C5—C6—C7—C8	−140.22 (15)	C8—N3—C13—C14	−75.4 (2)
N2—N3—C8—C7	−1.60 (15)	N3—C13—C14—C15	162.54 (15)
C13—N3—C8—C7	173.17 (13)	C13—C14—C15—C17	63.3 (2)
N2—N3—C8—C9	173.26 (13)	C13—C14—C15—C16	−173.43 (19)
C13—N3—C8—C9	−12.0 (2)		

### Hydrogen-bond geometry (Å, º)

**Table:**

*D*—H···*A*	*D*—H	H···*A*	*D*···*A*	*D*—H···*A*
C11—H11*B*···O2^i^	0.97	2.59	3.338 (2)	134

Symmetry code: (i) −*x*+3, −*y*+1, −*z*+1.
